# Development and *In Vivo* Characterization of Probiotic Lysate-Treated Chitosan Nanogel as a Novel Biocompatible Formulation for Wound Healing

**DOI:** 10.1155/2020/8868618

**Published:** 2020-12-28

**Authors:** Yousef Ashoori, Milad Mohkam, Reza Heidari, Seyedeh Narjes Abootalebi, Seyyed Mojtaba Mousavi, Seyyed Alireza Hashemi, Nasim Golkar, Ahmad Gholami

**Affiliations:** ^1^Biotechnology Research Center, Shiraz University of Medical Sciences, Shiraz, Iran; ^2^Department of Pharmaceutical Biotechnology, School of Pharmacy, Shiraz University of Medical Sciences, Shiraz, Iran; ^3^Student Research Committee, Shiraz University of Medical Sciences, Shiraz, Iran; ^4^Pharmaceutical Sciences Research Center, Shiraz University of Medical Sciences, Shiraz, Iran; ^5^Department of Chemical Engineering, National Taiwan University of Science and Technology, Taipei, Taiwan; ^6^Department of Mechanical Engineering, Center for Nanofibers and Nanotechnology, National University of Singapore, Singapore; ^7^Department of Pharmaceutics, School of Pharmacy, Shiraz University of Medical Sciences, Shiraz, Iran

## Abstract

Wound healing is a physiological reaction to tissue injuries which plays a crucial role in replacing the destroyed tissues. Probiotics produce valuable compounds that possess antibacterial and anti-inflammatory activities, immunomodulatory effects, and angiogenesis traits leading to the promotion of wound healing. Chitosan nanostructures have versatile properties making them quickly produced into gels, scaffolds, nanoparticles, beads, and sponge structures that can be incorporated into wound healing processes. In the current study, three formulations from nanogel consisting of probiotic supernatant (*Lactobacillus reuteri*, *Lactobacillus fermentum*, and *Bacillus subtilis* sp. *natto*)-loaded chitosan nanogels were prepared from the culture of corresponding cultures. The chitosan nanogels were previously characterized by Zetasizer, FTIR, and TEM. The prepared formulations' effectiveness and dressing activity were assessed by evaluating wound closure and histological trials in Sprague-Dawley rats. The results indicated that all probiotic lysate formulations have advantages over the wound healing process. However, Bacillus subtilis natto has a better wound healing quality, which is well known in pathology examination. The favorable effects of probiotic lysate nanogels, including the reasonable wound closing rate, good wound appearance, and satisfactory histological observation via in vivo examination, suggest it as a promising nominee for wound healing purposes.

## 1. Introduction

Wound healing, a network of complex biological processes involving many extracellular and intracellular macromolecules, plays a significant role in skin remodeling and reestablishing the skin barrier following damages [1, 2]. Management of the wound healing process costs billions of dollars per year directly, together with the time spent on its management. Although antibiotics are applied in modern wound management as routine care, they cannot cover all aspects of wound management [3]. Hence, seeking an alternative strategy that speeds the wound healing process is a noticeable research field for basic scientists and industrial experts.

Chitosan is a natural linear polysaccharide composed of poly (1, 4), B-D-glucopyranose amine units that can be obtained from the deacetylation of the crushing of crustacean chitin shells. This natural polymer has attractive features such as biologic activity, low toxicity, bioadhesive, biodegradability, biocompatibility, low cost, and antibacterial, antiviral, and antifungal properties [4, 5]. Chitosan nanogel (CS-NG) has been reported as a suitable natural biomedical polymeric nanosystem in wound healing [4]. They are superabsorbent of water and biological fluids to absorb more than 10–20% *w*/*w* distilled water. CS-NG also visualized a sound polymeric bioagent delivery system having a high drug loading capacity, stability, and switching property against physical changes, including ionic strength, pH, and temperature. Therefore, they have especially been recommended for various pharmaceutical applications, including drug delivery, antimicrobial activity, and wound healing [6, 7].

As live microorganisms that confer a health benefit on the host, probiotics can modulate the immune system, reduce inflammation, and promote wound healing processes [8]. Wound healing was also affected by the anti-inflammatory properties of extracellular polysaccharide (EPS) produced by probiotic bacteria. In the case of wounds, they also inhibited the proliferation of pathogens and showed antimicrobial properties [9, 10]. During the last century, a possible beneficial effect of probiotic bacteria on wound healing has been proposed via systemic induction of the immune response. Also, it is found that some certain probiotic strains may improve wound healing processes through the antibacterial, anti-inflammatory, and proteoglycan deposition, as well as angiogenesis traits [11, 12].

In the present study, a novel chitosan-based nanogel containing a probiotic supernatant complex was developed for skin delivery to promote the wound healing process. The prepared nanogels were characterized by their particle size, zeta potential, and morphology properties. The *in vivo* wound healing evaluations in the rat model were then performed, and the histological consequences have been assessed.

## 2. Materials and Methods

### 2.1. Materials

The chitosan was purchased from Katokichi Co., Japan. MRS (de Man, Rogosa, and Sharpe) broth, tryptic soy broth (TSB), and yeast extract were prepared from Difco (Detroit, USA). Maltose was purchased from Merck (C.A., USA).

### 2.2. Preparation of Probiotic Lysates

The probiotic bacteria of *Bacillus subtilis* sp. *natto* (*B.S.* natto, ATCC 15245), *Lactobacillus fermentum* (ATCC 9338), and *L. reuteri* (ATCC 23272) strains were used in the experiment.

The Lactobacilli bacteria were cultured using MRS (de Man, Rogosa, and Sharpe) broth medium (Difco, USA) for 48 h at 37°C under microaerophilic conditions until the stationary phase was achieved. *B.S.* natto was cultured using 10 g soy-peptone, 2 g potassium hydrogen phosphate, 1 g magnesium sulfate, 20 g maltose, 10 g yeast extract, and 2 g glucose at 37°C for 48 h. The pH was adjusted to 7.2 using acetic acid 0.2 M or sodium hydroxide 0.2 M. Inoculum of bacteria containing approximately 1 × 10^8^ to 10^9^ CFU/ml was prepared [13]. The number of viable bacteria was distinguished by plate counts using MRS agar (Difco, USA). The bacteria were then harvested by centrifugation (4000 rpm) for 20 min at refrigerator temperature. The harvesting supernatants were filtered through a 0.2 *μ*m membrane filter to remove the remaining bacteria and remains and then lyophilized before storage at −20°C. Bacterial counting on MRS agar plates showed no evidence of lactobacilli growth. The presence of lipopolysaccharide (LPS) in L.S. had been excluded with a diagnostic kit from Cambrex Corporation (East Rutherford, NJ).

### 2.3. Preparation of CS-NG

CS-NG were fabricated via ionic gelation of chitosan employing a polyanion, sodium triphosphate (TPP). In pleasing status, 1 ml of the polyanionic TPP solution (0.5%, *w*/*v*) was added to 7 ml of the chitosan suspension (0.06%, *w*/*v*) in acetate buffer (pH = 4) in a drop-wise manner, over a 2 min period under the continuous magnetic stirring (1500 rpm) at 28°C. Nanoparticles were obtained after centrifugation at 12,000 × g for 15 min. The supernatant was dropped, and the mass was resuspended in the deionized water [14].

### 2.4. Characterization of Nanogel

#### 2.4.1. Fourier Transform Infrared Spectroscopy (FT-IR)

The dried CS-NG was mixed with Potassium Bromide (KBr) powder and compressed into a tablet to perform Fourier transform infrared spectroscopy (FT-IR). The sample was scanned by the FT-IR spectrometer (6100 JASCO, Japan) using a resolution of 4 cm^−1^ and 64 coadded scans over the wavelength range of 400 to 4000 cm^–1^.

#### 2.4.2. Transmission Electron Microscopy (TEM)

The morphology and size of the prepared nanogels were determined using TEM. A total of 20 *μ*l of the sample was placed on a carbon film coated on 300 mesh copper grids (EMS) for 2 min, and the excess liquid was absorbed with a filter paper. The sample was then negatively stained with 20 *μ*l of 2% uranyl acetate solution for less than a minute, and the excess stain was absorbed again with a filter paper. The sample was allowed to air dry and examined using a Transmission Electron Microscope (EM10C, Zeiss, Germany) at an accelerating voltage of 100 kV.

#### 2.4.3. Particle Size and Zeta Potential

The particle size distribution, mean hydrodynamic diameter, polydispersity index (PDI), and zeta potential of the CS-NG were obtained by a Malvern Zetasizer (ZEN 3600, U.K.) at a fixed scattering angle (90°) and using a He-Ne laser (633 nm). The refractive index and viscosity of pure water were utilized for data analysis.

### 2.5. Preparation of Probiotic Lysate-Loaded Nanogels

One milligram of various lyophilized probiotic supernatants was individually added into 10 grams of the CS-NG (2.5%) for 5 minutes at room temperature to prepare different probiotic supernatant-enriched nanogels as tabulated in Table 1. Each probiotic supernatant-enriched nanogel formulation was then concentrated using the 30,000 MWCO Viva flow ultrafiltration kit, and the final concentration of 1% (*w*/*w*) was obtained.

### 2.6. Animal Study

In the present study, 25 mature Sprague-Dawley rats weighing 200-300 g were used. The rats were purchased from the Center of Comparative and Experimental Medicine at the Shiraz University of Medical Science and treated according to the discipline of animal management and welfare of the University of Helsinki, Finland. Animals were placed in a standard cage with free access to tap water and a rodent's pellet chow diet (RoyanFeed®, Isfahan, Iran). To eliminate any effects of stress conditions on the experimental rats, they were allowed to be adapted to the new laboratory conditions for 15 days. Then, the rats were divided into five experimental groups, as presented in Table 2. The experimental procedures were all performed according to the regulations of the Animal Ethics Committee of Shiraz University of Medical Sciences under code number I.R.SUMS.REC.1399.748.

The rats were anesthetized using a mixture of ketamine : xylazine 80 mg/kg : 10 mg/kg. Excision wounds of approximately 226 mm^2^ and 2 mm depth were made via cutting out of a skin layer from the shaved area. All medications in the form of topical formulations (each containing 1 g of probiotic lysate in 100 g of CS-NG (1% *w*/*w*)) were administered to the wounds every day. The contralateral injury in the same animal receiving CS-NG without any probiotic lysate and rats receiving no treatment served as a control group.

The percentage of wound healing was measured according to the following equation:

(1) $$\begin{array}{c} \text{Percentage of wound healing}=\left(1-\frac{\text{wound size in a specific day}}{\text{wound size in day }0}\right)\times 100. \end{array}$$

### 2.7. Wound Histopathological Analysis

After 14 days, the animals were sacrificed by spinal cord injury under anesthesia. Wound skin tissues (each in 3.5 cm × 1.2 cm) with full thickness were removed, and paraffin-embedded sections were prepared. The sections (each with 2 mm thickness) were cut with a microtome, cutting perpendicular to the width of the skin surface. The sections were all stained with hematoxylin-eosin. The degrees of epithelialization, fibrosis, inflammation, and granulation were assessed for all of the specimens. In order to calculate the wound healing process quantitatively, histopathological changes of skin tissue (the degrees of epithelialization, fibrosis, inflammation, and granulation) were blindly scored by an expert clinical pathologist as follows:

0: there is no apparent change in histopathological features of skin tissue

+1: mild changes could be diagnosed in histopathological features of skin tissue by the pathologist

+2: moderate changes could be diagnosed in histopathological features of skin tissue by the pathologist

+3: severe change could be diagnosed in histopathological features of skin tissue by the pathologist

### 2.8. Statistical Analysis

The data gathered from each group after three selected days were statistically analyzed by the Statistical Package for the Social Sciences (SPSS) software version 18.0 (SPSS Inc., USA) using analysis of variance (ANOVA) and Duncan's mean comparison test. Each group contained five animals, and a *P* value of less than 0.05 (*P* value < 0.05) was considered statistically significant. Results were stated as mean ± standard deviation (S.D.). Scores for tissue histopathological changes are represented as median and quartiles for five random pictures per group. The analysis of tissue histopathological alterations was performed by the Kruskal-Wallis followed by the Mann-Whitney *U* test.

## 3. Results and Discussion

### 3.1. CS-NG Characterization

**Figure 1:**
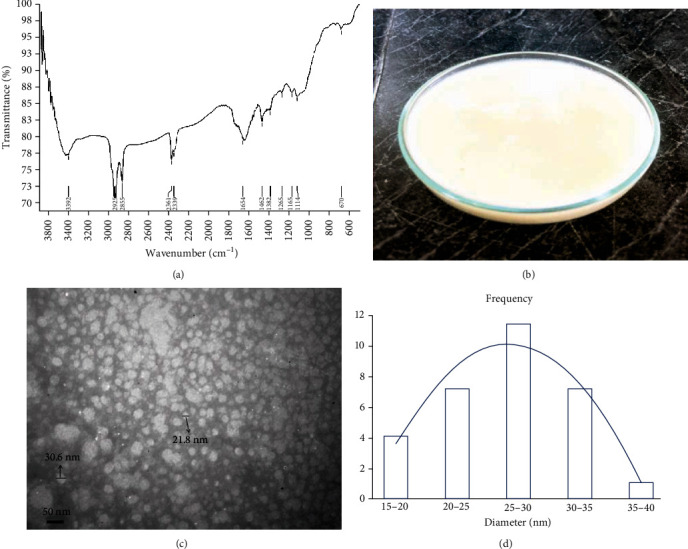
Characterization of chitosan nanogel: (a) FT-IR spectra, (b) appearance before formulation, (c) TEM image, and (d) mean size distribution.

The FT-IR spectra of CS-NG are shown in Figure 1(a). In this spectrum, there was a broad and strong peak at 3500-3300 cm^−1^, which corresponds to the overlapping of primary amine and hydroxyl groups stretching vibration. Also, deforming –NH2 vibration of hydroxyl peaks appeared at 1654 cm^−1^. The peak at 2925 cm^−1^ was attributed to the aliphatic carbon stretching vibrations. A characteristic peak at 1382 cm^−1^ was due to the C-N stretching vibration of amine groups, and the peak found at 1165 cm^−1^ belonged to the asymmetric stretch of the C-O-C bonds. Therefore, the FT-IR spectra showed the characteristics of CS-NG bonds, and it was concluded that the inter- and intramolecular bonds of nanogel were well formed. The synthesized chitosan was a smooth, uniform nanogel with semisolid consistency and white colour (Figure 1(b)). The pH of this nanogel was measured at 7.4.

The nanogel was characterized by TEM microscopy (Figure 1(c)). Based on the results obtained from TEM, chitosan nanoparticles had a spherical and uniform shape. According to TEM images, nanogels had an acceptable uniformity in size distribution with a size range of 10–50 nm. The average particle size of the chitosan nanogel was 25.6 ± 1.5 nm (Figure 1(d)).

The zeta potential measures the amount of electrostatic ability of multiagent disperse systems and calculates the rate of nanoparticle aggregation and agglomeration. Therefore, it can be considered an index representing the physical stability of multiagent disperse systems (Rachmawati et al., 2013). The zeta potential of nanogels was obtained as 13.07 ± 0.17 mV.

### 3.2. Wound Healing in Animal Studies

**Figure 2:**
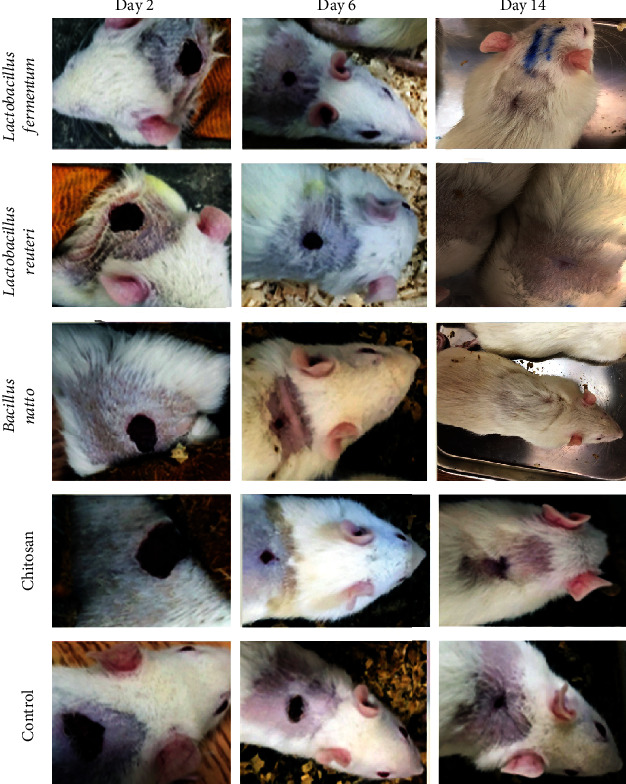
Photographs of the wound healing process in different groups: *Lactobacillus fermentum* supernatant-loaded chitosan nanogel as group 1, *Lactobacillus reuteri* supernatant-loaded chitosan nanogel as group 2, *Bacillus subtilis* sp. *natto* supernatant-loaded chitosan nanogel as group 3, chitosan nanogel without probiotic lysate as group 4, and the control group which receives no treatment as group 5. The photographs were taken after day 2, day 6, and day 14.

**Figure 3:**
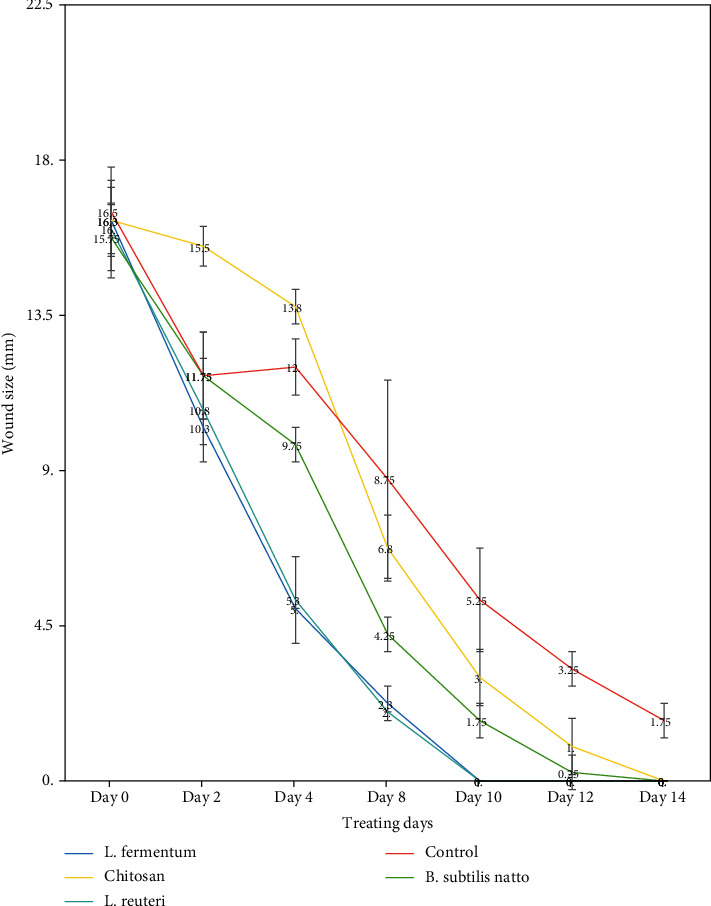
Effects of different groups on wound healing: *Lactobacillus fermentum* supernatant-loaded chitosan nanogel as group 1, *Lactobacillus reuteri* supernatant-loaded chitosan nanogel as group 2, *Bacillus subtilis* sp. *natto* supernatant-loaded chitosan nanogel as group 3, chitosan nanogel without probiotic lysate as group 4, and the control group which receives no treatment as group 5. Data are demonstrated as mean ± SEM (*n* = 6). ∗ indicates a significant difference from the fructose control group (*P* < 0.05).

The trend of wound healing during 14 days for topically treated rats by probiotic supernatants, chitosan, and untreated rats is shown in Figure 2. As illustrated in figures, a typical pattern of healing had been observed in all five groups. The trends related to the percent of wound healing in each experimental group are demonstrated in Figure 3. Results indicated that the wound size was averagely 16.5 ± 1.29 mm (which is considered 0% wound healing percent) on day 0 (the first day) for each rat in all the groups. The probiotic lysates and control groups had nearly the same size of wounds until day 2. Simultaneously, the CS-NG group had fewer wound healing rates. From the beginning, probiotic lysate groups showed a significant percentage of wound healing (*P* value < 0.05). The wound healing rates were higher in the groups receiving the *L. Fermentum* supernatant-loaded CS-NG and *L. reuteri* supernatant-loaded CS-NG than the other groups. The wound healing process in these two groups was completed by day 10. Even after 14 days (the end of the experiment), the healing process in the control group was not completed (Table 3). Khodaii et al. reported that administration of *L. reuteri* extract significantly promoted the wound healing processes by day 15, which is comparable with our results (the healing process was completed before ten days) [15]. In this regard, Han et al. claimed that *L. reuteri* enhanced wound healing via the PI3K/AKT/*β*-catenin/TGF*β*1 pathway [16]. As for *L. fermentum*, Brandi et al. stated that treatment of wounds with *L*. *fermentum* increases wound healing processes via exerting anti-inflammatory and antipathogenic influences [17]. Chitosan offers wound healing effects by stimulating the liberation of inflammatory cytokines, the proliferation of human skin fibroblasts and keratinocytes, and its antimicrobial activity [18]. However, the lonely chitosan administration in this study did not show a significant wound healing process compared to bacterial extract treatment, which indicated the higher activity of wound healing factors in these probiotic bacteria.

### 3.3. Histopathological Results

**Figure 4:**
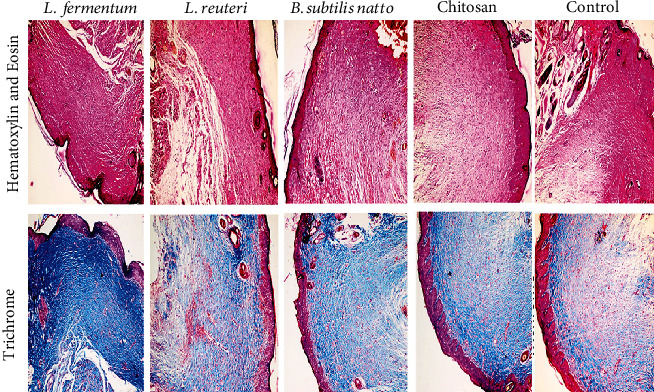
The histopathological examination in the different groups: *Lactobacillus fermentum* supernatant-loaded chitosan nanogel as group 1, *Lactobacillus reuteri* supernatant-loaded chitosan nanogel as group 2, *Bacillus subtilis* sp. *natto* supernatant-loaded chitosan nanogel as group 3, chitosan nanogel without probiotic lysate as group 4, and the control group which receives no treatment as group 5.

The histological assessment in rats offers essential facts for the situation of a wound. Skin tissue histopathological alterations are presented in Figure 4. Moreover, the scores of epithelization, inflammation, granulation, and fibrosis at the end of day 14 are summarized in Table 4. According to the results, the epithelization processes were complete in all groups except the control one at the end of day 14; in contrast, the inflammation in the control group was significantly high compared to all treated groups. The granulation of skin tissue was different between the various groups. Accordingly, chitosan and *B*.*S*. *natto* groups did not show granulation criteria, while *L. fermentum* and *L. reuteri* demonstrated a degree of inflammation. The highest inflammation was related to the control group. Although the degree of fibrosis was observed in all experimented groups, this factor is significantly higher in the control group than the others. The boosted effects of the probiotic supernatant-loaded CS-NG in wound healing may be attributed to their metabolites (including polysaccharides, lactic acid, acetic acid, and other chemicals), which provoke proteoglycan deposition and angiogenesis as well as reduction of inflammation along with invigorating various growth factors like EGF, PDGF, FGF, and TGF*β* [9, 10, 17–23].

In the past, researchers believed that probiotics were only useful in gastrointestinal problems and diseases [24]. Gradually, with more extensive research, the importance of probiotics in daily life becomes more apparent. The effects of different probiotic strains on hepatic steatosis, nonalcoholic fatty liver disease, diabetes, osteoporosis, and some other inflammatory diseases have been well studied in recent years [8, 11, 25]. In a recent study, the skin healing potential of the *Shewanella putrefaciens* on the wounded marine animal model was reported after dietary administration [26]. Due to the importance of accelerating and improving the quality of wound healing, probiotic lysate for the treatment of wounds is crucial. Besides, the use of CS-NG as a base for probiotic lysate increases the wound healing properties of the formulation. Multifunctional injectable three-dimensional nanostructured self-healing chitosan-based hydrogels and cryogels have been recently reported for promising conductivity and photothermal activity as a wound dressing for increasing the wound healing process [27–29]. This study showed promising results about wound healing potential of newly formulated probiotic lysates with chitosan nanogels. Recently, several studies have been published about the formulation of natural products with nanochitosan preparations as skin dressing specially in the injectable form [30]. In a study, Qu et al. reported that an injectable curcumin-based CS hydrogel preparation improved the wound healing process. The study revealed better granulation thickness and collagen disposition in the porcine skin soft tissues. They also showed a vascular endothelial growth factor upregulation in animal skin [31].

It should be noted that because of the effect of CS-NG together with probiotic lysates, our formulation may entail antimicrobial effects, and the pH regulation of wound local space avoids the adverse effects of broad-spectrum antibiotics. It seems that this formulation provided a three-dimensional network with desirable swellability for maintaining wound moisture and rheological properties to exhibit excellent wound healing capability. Further in-depth studies about various aspects of this formulation, the mechanisms involved in its effects, and more molecular information are needed before using this product for further applications. Accordingly, this novel formulation may open a new window on wound healing treatment in the future after deep consideration and clinical trials.

## 4. Conclusion

The chitosan was chosen as an intrinsically biocompatible and antimicrobial substance for further derivation to offer qualities to improve the antimicrobial activity and attain superb biocompatibility and good water maintenance nature. The FT-IR spectrum of nanogels pointed out that the inter- and intramolecular bonds of nanogel were well-formed. The morphological observation designates that the nanogels had an acceptable uniformity in size distribution with a size range of 10–50 nm and good physical stability of nanogels. Wound healing trials employing an animal model illustrated that incorporating the prepared novel probiotic lysate nanogel on to an open wound provokes a special wound closing rate compared to usual chitosan and gauze. In general, each of the probiotic lysate groups has advantages over the other groups. For example, the rate of wound closure in the group receiving lactobacilli is higher than that in the other groups. However, the group receiving *B*.*S*. natto has a better wound healing quality than other groups, which is well known in pathology examination. The reason for the slow wound closure rate in the group receiving supernatants of *B*.*S*. natto may be the presence of the enzyme nattokinase, which has anticoagulant properties [19]. All the resulted features offer that the prepared probiotic supernatant-loaded CS-NG can be regarded as an appropriate nominee in support of wound healing remedy along with its aspect for developing a novel outlook in nanomedicine.

## Figures and Tables

**Table 1:** Various probiotic-enriched CS-NG formulations.

Formulations	Composition
1	*Lactobacillus fermentum* supernatant-loaded chitosan nanogel
2	*Lactobacillus reuteri* supernatant-loaded chitosan nanogel
3	*Bacillus subtilis* sp. *natto* supernatant-loaded chitosan nanogel

**Table 2:** Various rat groups receiving different formulations.

Rat groups	Nanogel formulations
Group 1	*Lactobacillus fermentum* supernatant-loaded chitosan nanogel
Group 2	*Lactobacillus reuteri* supernatant-loaded chitosan nanogel
Group 3	*Bacillus subtilis* sp. *natto* supernatant-loaded chitosan nanogel
Group 4	Chitosan nanogel without probiotic lysate
Group 5	Control group which receives no treatment

**Table 3:** The percent of wound healing during 2 weeks of treatment with different probiotic supernatant-loaded chitosan nanogels.

	Day 2	Day 4	Day 8	Day 10	Day 12	Day 14
*L. fermentum*	39.71	70.59	86.77	100	100	100
*L. reuteri*	36.77	70.59	88.24	100	100	100
*B. subtilis natto*	30.89	42.65	75	89.71	98.53	100
Chitosan	8.9	19.12	60.30	82.36	94.12	100
Control	30.89	29.42	48.53	69.12	80.89	89.71

**Table 4:** Scores of skin histopathological changes in the animal model of wound healing.

Treatments	Epithelialization	Inflammation	Granulation	Fibrosis
Control	Incomplete	1 (2, 1)	3 (3, 2)	2 (3, 2)
Chitosan	Complete	0 (0, 0)^∗^	0 (0, 0)^∗^	1 (0, 1)^∗^
*Lactobacillus fermentum*	Complete	0 (0, 0)^∗^	1 (0, 1)^∗^	1 (0, 1)^∗^
*Lactobacillus reuteri*	Complete	0 (0, 0)^∗^	1 (0, 1)^∗^	1 (0, 1)^∗^
*Bacillus subtilis natto*	Complete	0 (0, 0)^∗^	0 (0, 0)^∗^	1 (0, 1)^∗^

Scores for tissue histopathological changes are represented as median and quartiles for five random pictures per group. ∗ indicates significantly different as compared with the control group (*P* < 0.05).

## Data Availability

The data used to support the findings of this study are included within the article.
